# Serum symmetric dimethylarginine in older dogs: Reference interval and comparison of a gold standard method with the ELISA

**DOI:** 10.1111/jvim.16981

**Published:** 2024-01-19

**Authors:** Sofie Marynissen, Greet Junius, Evi Van den Steen, Lisbeth Patteet, Luc Duchateau, Siska Croubels, Sylvie Daminet, Dominique Paepe

**Affiliations:** ^1^ Small Animal Department, Faculty of Veterinary Medicine Ghent University Merelbeke Belgium; ^2^ Sonic Healthcare Benelux Antwerpen Belgium; ^3^ Biometrics Research Group, Department of Veterinary and Biosciences, Faculty of Veterinary Medicine Ghent University Merelbeke Belgium; ^4^ Department of Pathobiology, Pharmacology and Zoological Medicine, Faculty of Veterinary Medicine Ghent University Merelbeke Belgium

**Keywords:** asymmetric dimethylarginine, canine, ELISA, SDMA

## Abstract

**Background:**

Serum symmetric dimethylarginine (SDMA) is used to screen for renal dysfunction in dogs. The gold standard technique for measuring SDMA, liquid chromatography‐tandem mass spectrometry (LC‐MS/MS) is not widely available. Age‐specific reference intervals for SDMA in older dogs are lacking.

**Objectives:**

Prospective study in older dogs to validate a commercially available LC‐MS/MS method for SDMA, compare SDMA concentrations with concentrations measured using ELISA and obtain a reference interval (RI) for older dogs using both methods.

**Animals:**

Client‐owned older dogs undergoing health screening.

**Methods:**

The LC‐MS/MS method was analytically validated (limit of detection, precision, and linearity). Serum was sent cooled overnight for ELISA or was frozen at −80°C until batch analysis using LC‐MS/MS. Results of LC‐MS/MS and ELISA were compared and RIs for older dogs were calculated according to international guidelines.

**Results:**

The LC‐MS/MS method showed good linearity (*r* ^2^ = .99) and precision (coefficient of variation <10%), with a laboratory RI between 8.0 and 14.0 μg/dL. Paired measurements were available from 118 different dogs. Median SDMA concentration were 9.4 (range, 5.0‐21.2) using LC‐MS/MS and 12.0 (range, 5.0‐22.0) μg/dL using ELISA. Both methods significantly differed with a mean difference of 2.2 μg/dL. The RI for older dogs for LC‐MS/MS was 4.4‐15.0 μg/dL, and for ELISA was 6.4‐17.4 μg/dL.

**Conclusions and Clinical Importance:**

The ELISA provided significantly higher SDMA concentrations compared to the validated LC‐MS/MS method, indicating the need for device‐ or assay‐specific RI. The obtained age‐specific RI for SDMA is considerably higher in older dogs compared to the general laboratory RI.

**Abbreviations**
ADMA: asymmetric dimethylarginine
CSLI: Clinical and Laboratory Standards Institute
CV: coefficient of variation
LC‐MS/MS: liquid chromatography‐tandem mass spectrometry
LOD: limit of detection
PECCT: plasma exogenous creatinine clearance time
PSI: pounds per square inch
RI: reference interval
SDMA: symmetric dimethylarginine
UP: ultrapure water
USG: urine specific gravity
WSAVA: World Small Animal Veterinary Association

## 1 INTRODUCTION

Early detection of acute or chronic renal dysfunction is considered crucial, both in human and veterinary medicine. Early diagnosis allows timely preventive measures to be taken, as well as institution of adequate treatment to improve outcome.^1^, ^2^ Serum symmetric dimethylarginine (SDMA) concentration appears to be of added value combined with serum creatinine concentration to screen for renal dysfunction in dogs and cats.^1^, ^3^, ^4^, ^5^ In initial reports, liquid chromatography‐tandem mass spectrometry (LC‐MS/MS) was used to quantify SDMA in serum.^5^, ^6^ The LC‐MS/MS method is considered the gold standard when measuring SDMA in small animals, but it is less widely available than the routinely used ELISA from IDEXX laboratories (IDEXX SDMA EIA test, IDEXX BioAnalytics, Vet Med Labor GmbH, Kornwestheim, Germany). Both methods are equal in workload and rapidity, although the needed technology is more widely available for ELISA.^7^ In recent studies, IDEXX SDMA performed similarly compared to serum creatinine concentration to detect decreased glomerular filtration rate (GFR) in both dogs and cats.^8^, ^9^ In the study of dogs, the cutoff for an optimal combination of sensitivity and specificity to detect a decreased GFR was 16 μg/dL.^8^ In the study of cats, a cutoff of 19 μg/dL was suggested.^9^ Another recent study in dogs suggested 18 μg/dL as cutoff for detection of ≥40% decrease in GFR (sensitivity, 90%; specificity, 83%).^10^ These results are substantially higher than the upper reference limit (>14 μg/dL) of the IDEXX SDMA. Studies found a high analytical variability for IDEXX SDMA (13.5%, after proprietary adjustments improved to 6.2%) in cats.^11^, ^12^ A comparative study in dogs found a high bias (>11%) between a point‐of‐care analyzer (IDEXX Catalyst SDMA Test) and the commercial laboratory IDEXX SDMA test.^13^ Biological variability reported over a 1.5 year period was rather small (14%) in dogs.^14^ Furthermore, the effect of interfering substances (eg, hemolysis, lipemia, bilirubin, asymmetric dimethylarginine [ADMA]) on SDMA measurement previously was tested for LC‐MS/MS.^15^ Although it is stated that the IDEXX SDMA test is not affected by mild to moderate hemolysis or any degree of lipemia or icterus,^15^, ^16^ these data have, to our knowledge, not been published in the peer‐reviewed literature. Thus, further research exploring the reasons for the high analytic variability of IDEXX SDMA is warranted.

Age‐specific reference intervals (RIs) are well established for young, growing small animals.^17^, ^18^, ^19^ Health screening studies have emphasized the need for age‐specific RIs in older dogs and cats as well.^20^, ^21^ Age‐specific RIs are important, because aging can result in several physiological changes, with age‐related but clinically unimportant changes.^22^ Currently, the IDEXX SDMA method only has an age‐specific RI for puppies (range, 0‐16 μg/dL), but not for older dogs.

Our prospective study aimed at first to validate a LC‐MS/MS method, that is routinely performed in a commercial laboratory (Sonic Healthcare Benelux, Antwerpen, Belgium), for serum SDMA and ADMA measurement in dogs. Secondly, we aimed to compare SDMA results using the LC‐MS/MS with the IDEXX SDMA test using an ELISA (IDEXX BioAnalytics, Vet Med Labor GmbH, Kornwestheim, Germany), and assess the influence of hemolysis, lipemia and ADMA concentrations on the difference between both methods. Thirdly, for both methods, an age‐specific RI was calculated for older dogs.

## 2 MATERIALS AND METHODS

### 2.1 Study population

Serum samples of older dogs, as defined based on a previously published human and pet analogy chart^23^ (Table 1), were used. Weight categories were slightly adapted to avoid exclusion of certain weights (eg, 9.2 kg). All dogs were prospectively recruited for complete health screening (blood pressure measurement, physical examination, CBC, serum biochemistry profile, urinalysis) as part of an ongoing longitudinal study. The study protocol was based on previously published health studies.^21^, ^23^ To be included in the longitudinal study, dogs had to be healthy based on owner observation, meaning that, in the owner's opinion, the dog did not have any problem necessitating veterinary care. Preventive medication (eg, anti ecto‐ and endoparasitic drugs, vaccination) was allowed until 2 weeks before health assessments, other treatment was not allowed during the 2 months preceding inclusion. During the 2‐year follow‐up, medical or surgical treatment was allowed. The study was completed at the Small Animal Clinic, Ghent University, between July 2019 and April 2021. All dogs were privately owned, the owners signed an informed consent form, and the study was approved by the local and national ethical committees (EC 2019/39, DWZ/EV/19/115/75).

**TABLE 1:** Descriptive data expressed in median (range) of 121 older dogs included in the study.

		0 to ≤9.1 kg	>9.1 to ≤22.7 kg	>22.7 to ≤54.5 kg	>54.5 kg
	Global study population	(8 years)	(7 years)	(6 years)	(4 years)
Number	121	39	36	44	2
Age (years)	10.1 (5.1‐16.6)	10.7 (7.1‐16.6)	9.9 (6.1‐15.5)	9.65 (6.1‐15.1)	7.1 (5.1‐9.1)
Weight (kg)	16.4 (1.2‐73.8)	6.1 (1.2‐8.8)	15 (9.2‐22.6)	30.2 (23.5‐40.7)	66.4 (59‐73.8)
BCS (/9)	5 (3‐8)	5 (3‐7)	5 (4‐8)	5 (3‐8)	5 (6‐7)
MCS (/4)	1 (1‐4)	1 (1‐2)	1 (1‐4)	1 (1‐3)	1 (no range)
Creatinine (μmol/L)	90 (40‐171)	77 (44‐139)	97 (47‐171)	96.5 (63‐156)	111 (100‐112)
SDMA (LC‐MS/MS, μg/DL)	9.4 (5.0‐21.2)	10.7 (5.4‐16.7)	9.0 (5.0‐21.2)	9.2 (5.1‐16)	9.3 (7.3‐11.2)
SDMA (ELISA, μg/dL)	12.0 (5.0‐22.0)	12.0 (7.0‐19.0)	11.0 (8.0‐22.0)	11.0 (5.0‐20.0)	10.5 (10.0‐11.0)
USG	1.032 (1.009‐1.055)	1.032 (1.011‐1.055)	1.035 (1.011‐1.055)	1.030 (1.011‐1.055)	1.009 (1 missing value)

*Note*: Data are presented for the global study population, and for the 4 weight categories used for inclusion of the dogs. Based on an adapted version of a previously published human/pet analogy chart,^19^, ^21^ a minimum age per weight class (visible as a subtitle per weight class) was intended. In the weight class <9.1 kg and >9.1 to <22.7 kg, there were each 2 dogs with an age below the target age.

Abbreviations: BCS, WSAVA body condition score; LC‐MS/MS, liquid chromatography‐tandem mass spectrometry; MCS, WSAVA muscle condition score; SDMA, symmetric dimethyl arginine; USG, urine specific gravity.

In a subgroup of the included dogs, plasma exogenous creatinine clearance time (PECCT) test was performed. Briefly, dogs were brought in by their owners in the morning, fasted overnight, with free access to water. A creatinine solution (40 mg/kg body weight of an 80 mg/mL solution) was injected via a cephalic catheter. Blood was collected from the jugular vein before and at 5, 15, 60, 120, 240, 360, and 480 minutes after injection in EDTA tubes. During the test, dogs were kept fasted and allowed access to water. Samples were centrifuged within 2 hours and stored in aliquots of 500 μL at −80°C until assayed. Frozen samples were sent in batch for creatinine analysis (kinetic test, Beckmann Coulter, Suarlee, Belgium). Individual plasma data were subjected to noncompartmental analysis for clearance calculation using a software program (Royal Canin creatinine clearance calculator, 2003).^24^, ^25^ The plasma clearance of creatinine was determined by dividing the actual administered dose of creatinine by the corresponding area under the plasma concentration vs time curve (AUC), and indexed to body weight (mL/min/kg). Previously described, weight categories‐based RIs (Lefebvre HP, Jeunesse E, Concordet D et al. Assessment of glomerular filtration rate using plasma exogenous creatinine clearance test: preliminary results in a healthy canine population. J Vet Intern Med. 2004;415 [abstract]): mini, 3.7 ± 0.5 mL/min/kg; medium, 3.0 ± 0.5 mL/min/kg; maxi, 2.5 ± 0.4 mL/min/kg and giant, 2.4 ± 0.6 mL/min/kg breed values were used.

### 2.2 Analytical validation of LC‐MS/MS method for SDMA and ADMA

Symmetric and asymmetric dimethylarginine concentrations were determined using a LC‐MS/MS method that is routinely performed in a commercial laboratory (Sonic Healthcare Benelux, Antwerpen, Belgium). In brief, 50 μL of serum, 100 μL of internal standard working solution (SDMA‐D6/ADMA‐D6, 50 μg/L, Toronto research chemicals INC, Toronto, Canada) and 200 μL of 1 M HCl in butanol were added to centrifuge tubes. A derivatization step was performed by adding 1M HCI in 1‐butanol and placing the samples for 30 minutes at 65°C. Supernatant was transferred to vials and 10 μL was injected into the high‐performance liquid chromatography (HPLC; AB Sciex LLC, Framingham, United States) equipped with a C18 column (Phenomenex C18 XB, Utrecht, Netherlands; 100 × 4.6 mm, 2.6 μm). Components were eluted using a gradient of 0.1% formic acid in ultrapure water (UP) and 0.1% formic acid in acetonitrile at a flow rate of 0.6 mL/min. A triple quadrupole MS instrument, type SCIEX 5500 (AB Sciex LLC, Framingham, United States), was used in positive electrospray ionization mode and multiple reaction monitoring detection. The used transitions were *m/z* 259.1 → 227.9 and 259.1 → 189.0 for SDMA, 265.1 → 231.0 for SDMA‐d6, 259.1 → 214.0 and 259.1 → 112.0 for ADMA, and 265.3 → 214.3 for ADMA‐d6. The following source parameters were used: temperature, 650°C; curtain gas, 40 pounds per square inch (psi); ion source gas 1, 40 psi; ion source gas 2, 40 psi; ion spray voltage, 1500 V and collision gas, 8 psi.

The LC‐MS/MS method was analytically validated to measure SDMA and ADMA in canine serum according to Belgian Accreditation Criteria (BELAC; ISO15189)^26^ by determining the limit of detection (LOD), precision, linearity and carry over.^27^ Limit of detection was calculated using the formula $\frac{\sqrt{2{\left(\mathrm{SD}\right)}^{2}}}{a}\times 3$ based on the results of 10 replicate measurements of a standard solution with a concentration of 1.35 μg/dL, with “SD” being the standard deviation and “a” being the slope of the curve between the concentration and the area. Assay imprecision was evaluated by measuring the intra‐ and interassay coefficient of variation (CV). A serum pool was measured 20 times within 1 run to determine intra‐assay variation. The intra‐assay CV was determined by dividing the SD of the parallel measurements by their mean and multiplying by 100. The interassay CV was determined similarly from the measurements on 20 consecutive days. To test linearity, a standard curve in UP was prepared, ranging from 6.5 to 259.7 μg/dL for SDMA and from 9.6 to 771.3 μg/dL for ADMA. An F test was used to determine if a significant difference existed between the SD of the y residuals of the quadratic and linear curves. Carry‐over was assessed by analyzing 3 blank samples (mean) following the calibration standard sample (SDMA [1000 μg/L] or ADMA [1500 μg/L]), using the criterium <LOD. The RI for the overall dog population for SDMA was based on previous LC‐MS/MS studies^15^ (Rentko V, Nabity M, Yerramilli M, et al. Determination of serum symmetric dimethylarginine reference limit in clinically healthy dogs. [ACVIM abstract P‐7]. J Vet Intern Med. 2013;27:750), and confirmed to be between 0 and 14 μg/dL in a large group (n = 239) of healthy appearing, adult dogs (age range, 1‐8 years) with serum creatinine concentration within RI (<75 μmol/L + body weight) divided over 4 weight groups (<10 kg [n = 58], 10‐20 kg [n = 53], 20‐30 kg [n = 60], 30‐40 kg [n = 33] and > 40 kg [n = 35]). The laboratory RI for ADMA was set, based on a group of healthy appearing dogs (n = 40; median age, 4 years [range, 0.5‐12.2]; median weight, 19.5 kg [range, 4.9‐65]), without abnormalities on routine blood tests (CBC, serum biochemistry profile, preprandial bile acids concentrations).

### 2.3 Method comparison for SDMA

All dogs were fasted overnight, and allowed free access to water. Ten milliliters blood was taken from the jugular vein. To obtain serum, coagulated tubes were centrifuged within 30 minutes at 1372 × *g* and serum was divided into aliquots of 300 μL. One aliquot was transported cooled (4°C) overnight for the IDEXX SDMA method (IDEXX BioAnalytics, Vet Med Labor GmbH, Kornwestheim, Germany), the remaining aliquots were stored at −80°C. Henceforth, IDEXX SDMA will be referred to as ELISA. For LC‐MS/MS (SDMA and ADMA) frozen serum samples were transported at −20°C in 3 batches (Sonic Healthcare Benelux, Antwerpen, Belgium). Absence or presence of lipemia or hemolysis in the serum sample was noted. Both were assessed using photometry with subsequent conversion into semiquantitative data (score 1 = absence of hemolysis/lipemia − score 4 = severe hemolysis/lipemia;IDEXX BioAnalytics, Vet Med Labor GmbH, Kornwestheim, Germany).

### 2.4 Age‐specific reference interval

An age‐specific SDMA RI for older dogs was defined following the guidelines of the American Society of Veterinary Clinical Pathology (ASVCP).^28^ To do so, SDMA measurements of both methods from older dogs without renal azotemia were used and we aimed to include at least 120 dogs. Renal azotemia was defined as serum creatinine concentration (enzymatic assay) exceeding the upper end of the RI (>1.8 mg/dL or >159 μmol/L; Catalyst Dx Chemistry Analyzer, IDEXX Laboratories Inc, United States) in combination with inadequately concentrated urine (USG <1.030; Master refractometer, ATAGO CO. LTD, Tokyo, Japan). Serum samples of dogs with a serum creatinine concentration >1.8 mg/dL and adequately concentrated urine (USG ≥1.030) were eligible for inclusion. The World Small Animal Veterinary Association (WSAVA) body condition score (BCS) and muscle condition score (MCS) were noted.^29^, ^30^ The age‐specific 95% RI was obtained using the normal distribution assumption limits according to the Clinical and Laboratory Standards Institute (CSLI) guidelines (sample size between 40 and 120 subjects), including the 90% confidence interval for the reference limits as described previously for both methods.^28^

### 2.5 Statistical analysis

Statistical analysis was performed using the statistical software package R (R module of SAS version 9.3, SAS Institute, NC, United States). Differences in SDMA results between the 2 methods (ELISA and LC‐MS/MS) were analyzed using a nonparametric Wilcoxon rank sum test. A Bland‐Altman plot was constructed to more thoroughly evaluate the agreement between both SDMA methods.^31^ The nonparametric Spearman rank correlation coefficient was derived to assess the association between the grade of hemolysis or lipemia, ADMA concentration and PECCT results on one hand, and the difference between the SDMA results between the 2 methods on the other hand. The level of significance was set at 5%.

## 3 RESULTS

### 3.1 Study population

Serum samples of 121 dogs were available for the study. Descriptive data are shown in Table 1. The study population comprised 11 mixed breed dogs, 10 Border Collies, 9 Belgian Shepherds, 8 Golden Retrievers, 8 Chihuahuas, 6 Labrador Retrievers, 6 Dachshunds, 5 Shetland Sheepdogs, 5 Jack Russell Terriers, 5 Cavalier King Charles Spaniels, 4 Shih Tzus, 4 English Cocker Spaniels, 4 Rottweilers and ≤3 dogs of 28 other breeds. Six dogs were on (intermittent) drug treatment at the time of sampling (meloxicam, n = 3; grapiprant, n = 1; benazepril, n = 1; prednisolone, n = 1). One other dog underwent radiation therapy for a Stage 3 anal sac adenocarcinoma previously diagnosed in the longitudinal study. In 14 dogs, PECCT measurement was available and for all dogs the GFR was within previously described reference intervals (Lefebvre HP, Jeunesse E, Concordet D et al. Assessment of glomerular filtration rate using plasma exogenous creatinine clearance test: preliminary results in a healthy canine population. J Vet Intern Med. 2004;415 [abstract]). Descriptive data of these dogs are shown in Table 2; none of these dogs were on medical treatment. Median time between sampling for SDMA measurement and performing PECCT was 0 (range, 0‐6) weeks. Median storage time until serum creatinine concentration measurement was 5.5 (range, 1.2‐13.1) months.

**TABLE 2:** Descriptive data expressed as median (range) of 14 older dogs in which estimation of the glomerular filtration rate was performed.

Age (years)	12.1 (6.1‐15.1)
Weight (years)	23.8 (12.5‐39.5)
Creatinine (μmol/L)	82 (47‐161)
BCS (/9)	5.5 (3‐7)
MCS (/4)	1 (1‐3)
SDMA (LC‐MS/MS, μg/dL)	8.9 (5.0‐18.4)
SDMA (ELISA, μg/dL)	11 (7‐20)
USG	1.026 (1.015‐1.036)
PECCT (mL/min/kg)	3.4 (2.4‐3.9)

*Note*: One dog had a serum creatinine concentration above the reference limit (>159 μmol/L), 1 other dog had a SDMA concentration above the reference limit (>14 μg/dL) for both methods. In both cases USG was ≥1.030.

Abbreviations: BCS, WSAVA body condition score; LC‐MS/MS, liquid chromatography‐tandem mass spectrometry; MCS, WSAVA muscle condition score; PECCT, plasma exogeneous creatinine clearance test; SDMA, symmetric dimethyl arginine; USG, urine specific gravity.

### 3.2 Analytical validation of LC‐MS/MS for SDMA and ADMA

The LOD for SDMA was 0.4 μg/dL. The SDMA intra‐ and interassay CV were <10%, with a good linearity (*r* ^2^ = .9997). The RI was 8‐14 μg/dL. Similarly, for ADMA measurement, good precision (CV < 10%) and linearity (*r* ^2^ = .9996) was obtained. The LOD for ADMA was 0.4 μg/dL and the RI was 18.1‐48.5 μg/dL. Negligible carry‐over was observed.

### 3.3 Method comparison for SDMA

**FIGURE 1:**
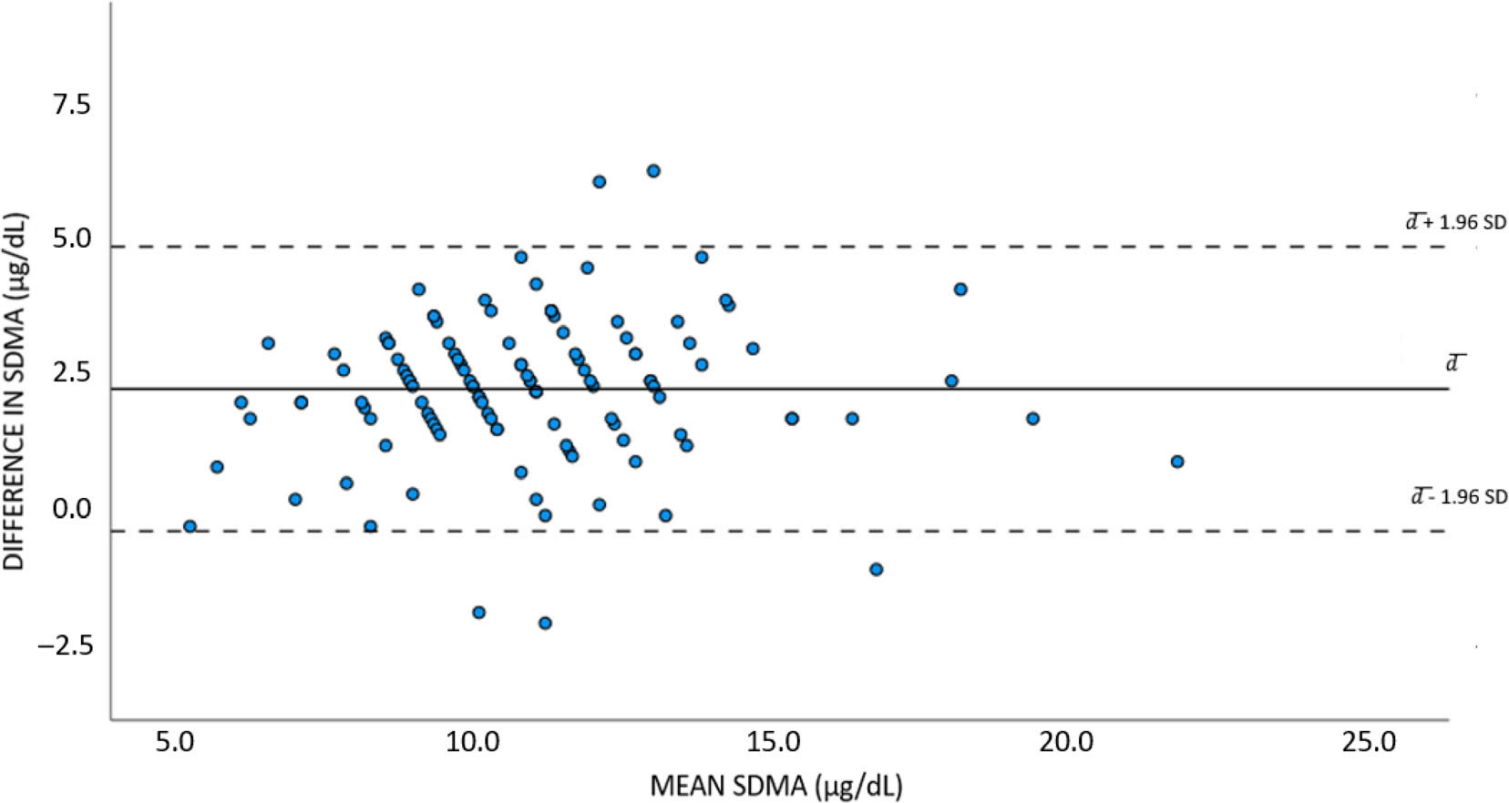
Bland‐Altman plot of 118 paired SDMA measurements, with ELISA and LC‐MS/MS method. The differences between paired measurements (SDMA ELISA − SDMA LC‐MS/MS; DIFFERENCE IN SDMA, y‐axis) are plotted against their means (MEAN SDMA, x‐axis). The solid black horizontal line represents the mean difference ($\bar{d}$) and shows that the average difference in SDMA between both methods is 2.2 μg/dL. The dashed horizontal lines describe the 95% limits of agreement (95% LoA) and are computed as the mean difference ±1.96 × SD of the difference. LC‐MS/MS, liquid chromatography‐tandem mass spectrometry; SDMA, symmetric dimethylarginine.

Median storage time until LC‐MS/MS analysis was 3 (range, 1‐19) months, all measurements using this method were performed between February and July 2021. In 3 samples, SDMA could not be assessed by ELISA, because of interfering lipemia or hemolysis (n = 2, score 4 lipemia; n = 1, no score for lipemia given; n = 1, score 4 hemolysis; n = 2, no score for hemolysis given), and thus 118 duplicate measurements were available for statistical analysis. Median SDMA was 9.4 (range, 5‐21.2) for LC‐MS/MS and 12 (range, 5‐22) μg/dL for the ELISA method. A significant difference (*P* < .05) was found between the 2 methods. The mean difference equaled 2.2 μg/dL (95% confidence interval [CI], 1.9‐2.4). The Bland‐Altman plot (Figure 1) indicated a systematic bias, with higher SDMA results for the ELISA method compared with LC‐MS/MS, but a change in variation with increasing SDMA concentrations was not observed. In 8% (9/118) of the dogs, SDMA was above the upper laboratory RI (>14 μg/dL) for ELISA, but still within the laboratory RI for LC‐MS/MS.

**FIGURE 2:**
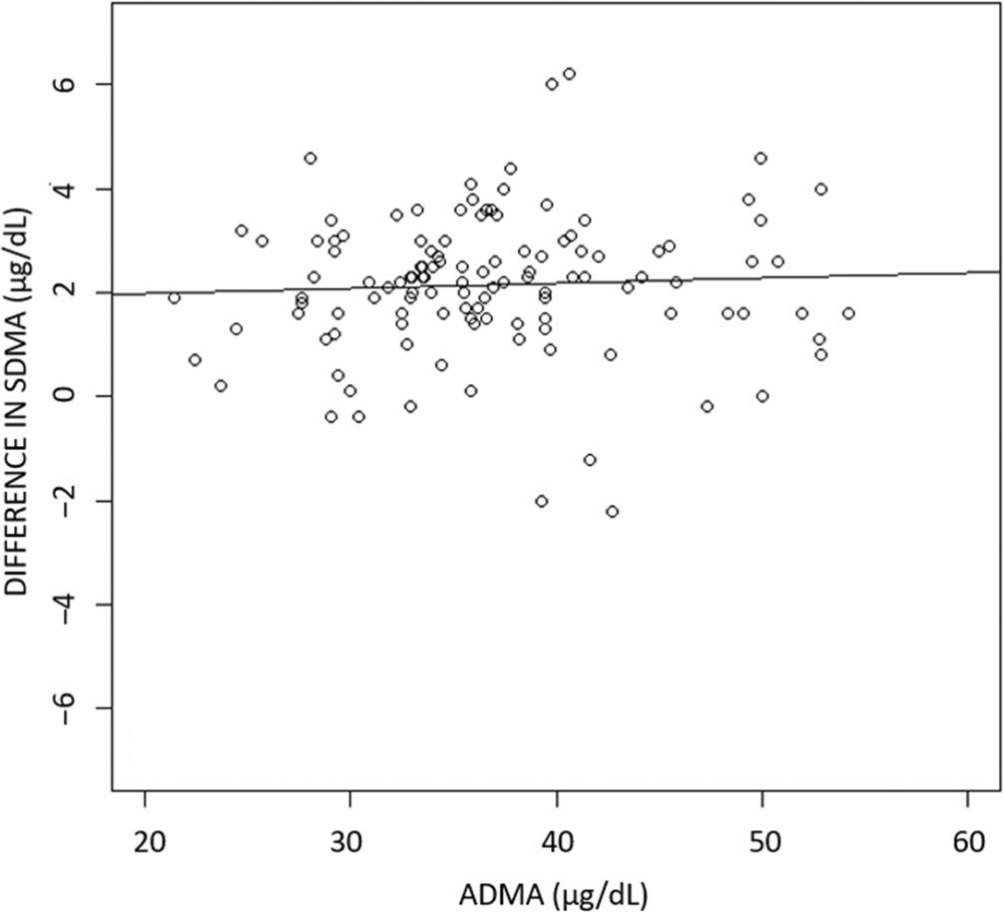
Scatter plot to graphically represent the effect of the ADMA concentration on the difference in obtained SDMA concentrations in 118 paired samples from dogs. Each circle represents the difference between SDMA ELISA and SDMA LC‐MS/MS (DIFFERENCE IN SDMA, y‐axis) and the ADMA concentration (ADMA, x‐axis) for 1 dog. The drawn line represents the weak observed positive relationship (slope = 0.00942; *P* > .05). ADMA, asymmetric dimethylarginine; LC‐MS/MS, liquid chromatography‐tandem mass spectrometry; SDMA, symmetric dimethylarginine.

**FIGURE 3:**
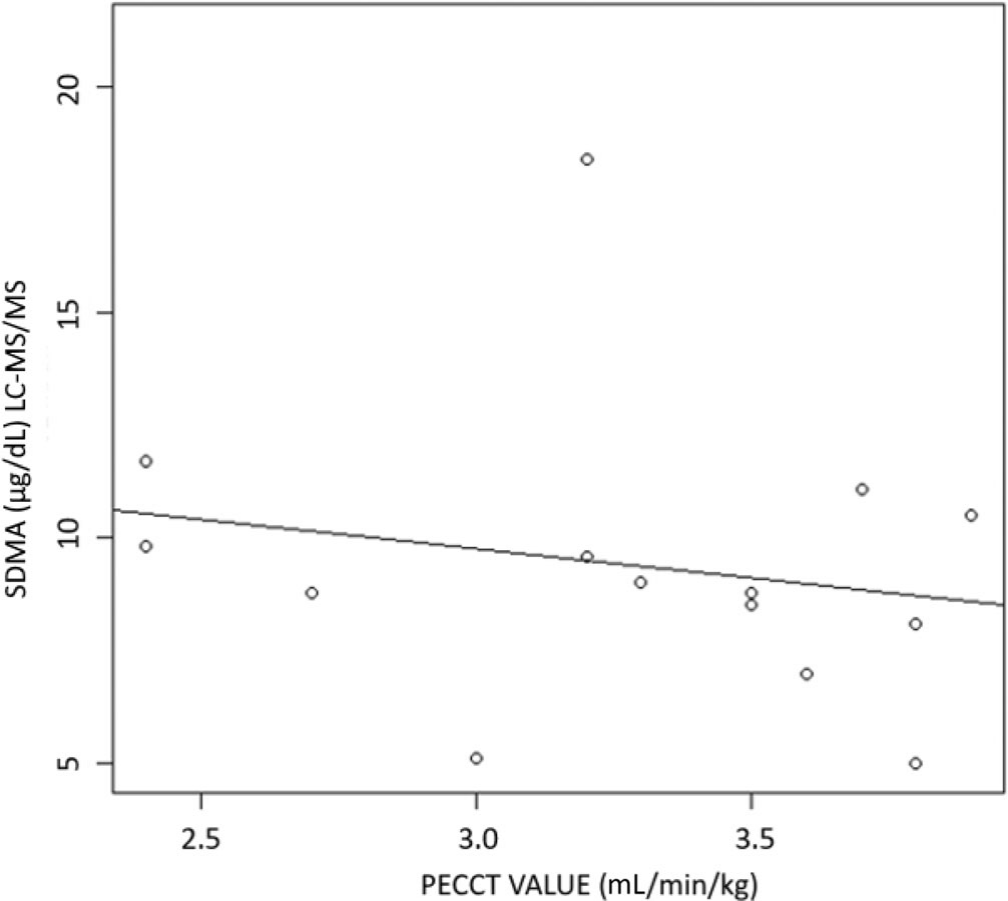
Scatter plot to graphically represent the effect of the PECCT value (proxy for the glomerular filtration rate) on the SDMA concentration for 14 dogs. The drawn line represents the weak observed negative relationship (slope = −1.292; *P* > .05). LC‐MS/MS, liquid chromatography‐tandem mass spectrometry; PECCT, plasma exogeneous creatinine clearance test; SDMA, symmetric dimethylarginine.

Median ADMA determined by LC‐MS/MS was 35.8 (range, 21.4‐54.2) μg/dL. A weak positive (slope, 0.08) relationship was found between the ADMA concentration and the difference in SDMA between both methods, but this did not differ significantly from zero (*P* > .05; Figure 2). A weak negative (slope, −0.29), nonsignificant (*P* > .05) relationship was found between calculated GFR and the LC/MS‐MS SDMA result (Figure 3).

**FIGURE 4:**
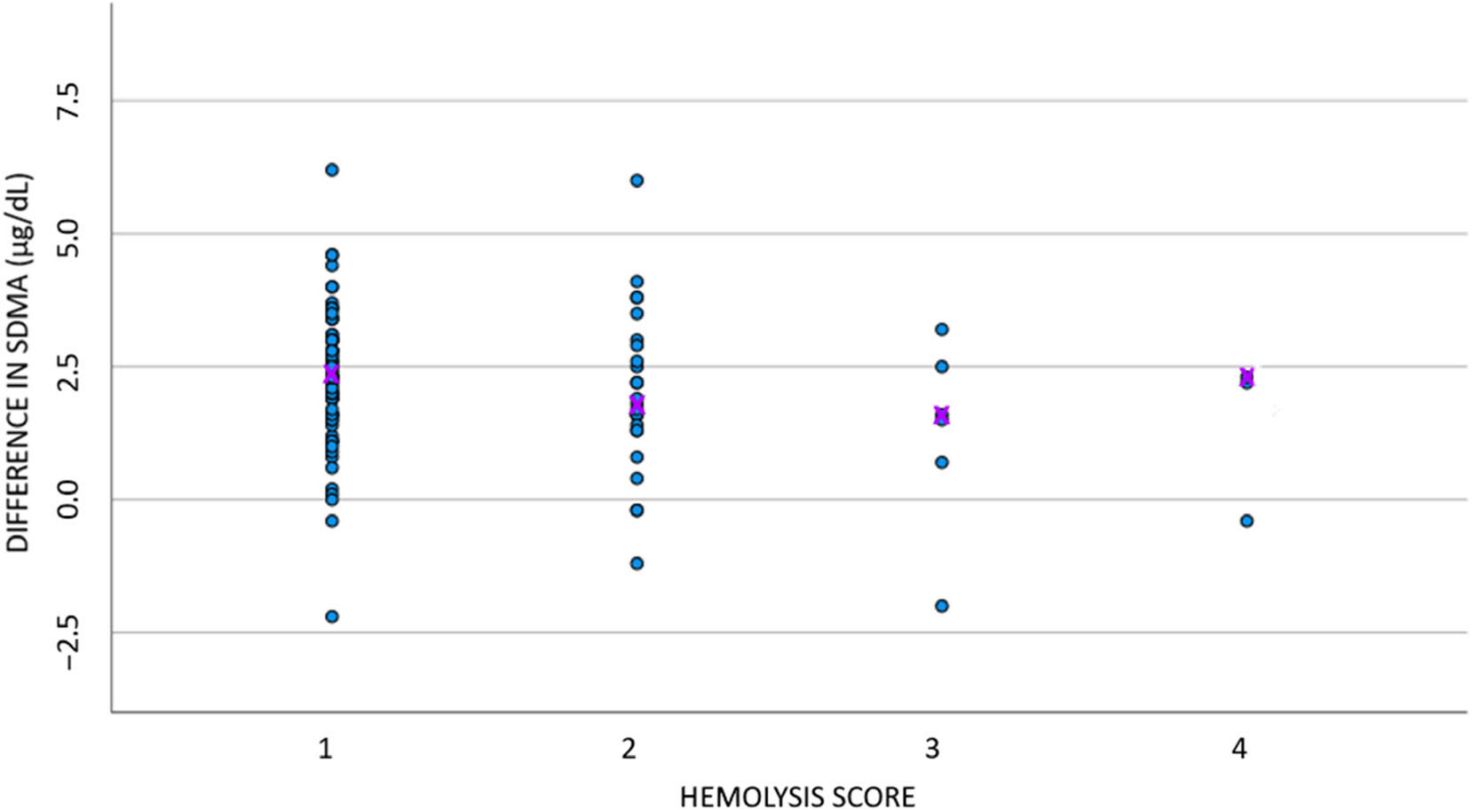
Scatter plot to graphically represent the effect of hemolysis for 121 dogs (x‐axis; score 1: absence of hemolysis [n = 76]; score 2: mild hemolysis [n = 26]; score 3: moderate hemolysis [n = 6]; score 4: severe hemolysis [n = 4]) on the difference (SDMA ELISA − SDMA LC/MS‐MS) between the 2 SDMA methods (y‐axis). In 9 dogs the hemolysis score was not available. The purple x indicates the median concentration per score. LC‐MS/MS, liquid chromatography‐tandem mass spectrometry; SDMA, symmetric dimethylarginine.

**FIGURE 5:**
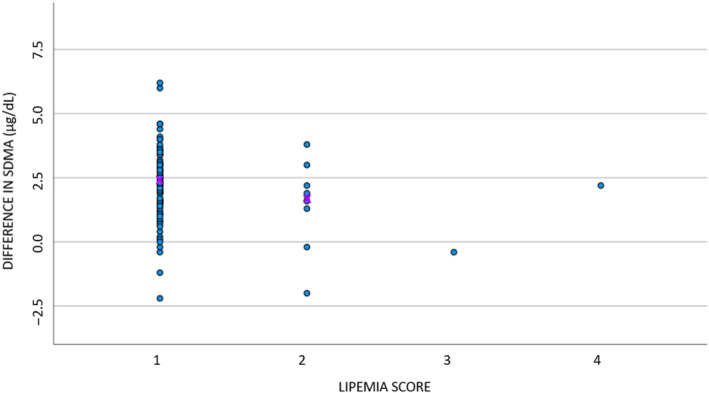
Scatter plot to graphically represent the effect of lipemia for 121 dogs (x‐axis; score 1: absence of lipemia [n = 99]; score 2: mild lipemia [n = 10]; score 3: moderate lipemia [n = 1]; score 4: severe lipemia [n = 3], no score determinable in n = 8) on the difference (SDMA ELISA − SDMA LC/MS‐MS) between the 2 SDMA methods (y‐axis). The purple x indicates the median value per score. LC‐MS/MS, liquid chromatography‐tandem mass spectrometry; SDMA, symmetric dimethylarginine.

The difference in SDMA between the 2 methods in the absence of hemolysis (score 1) equaled 2.3 (SD, 0.15). With increasing presence of hemolysis, the difference between the 2 methods decreased, with a decrease of −0.38 (SD, 0.18) with each unit increase in hemolysis score. This slope differed significantly from zero (*P* < .05; Figure 4). No association could be demonstrated between presence of lipemia and the difference between the 2 methods (*r* = −.18; *P* > .05; Figure 5).

### 3.4 Age‐specific reference interval

**FIGURE 6:**
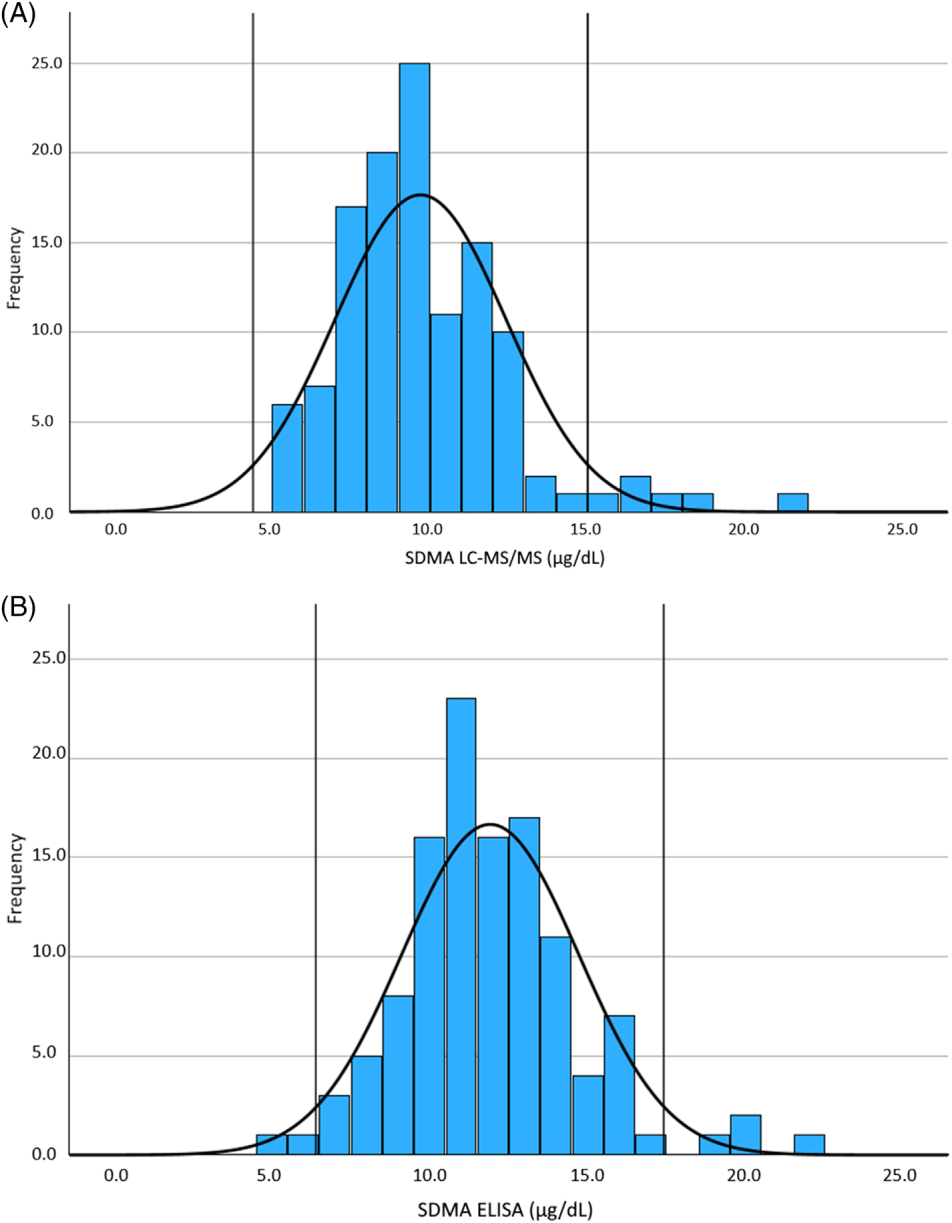
Histogram representation of an age‐specific reference interval obtained in 120 older dogs without renal azotemia (ie, serum creatinine concentration >159 μmol/L and USG <1.030), for LC‐MS/MS method (A) and ELISA method (B), respectively. The 2 long vertical lines correspond to the lower and upper limit of the 95% reference interval. Note that because of the small sample size, reported limits of the 95% reference interval might be variable. Therefore, also the 90% CI of the limits are provided, and given by 4.4 (90% CI, 3.7‐5.1) to 15.0 (90% CI, 14.3‐15.7) μg/dL and 6.4 (90% CI, 5.7‐7.2) to 17.4 (90% CI, 16.7‐18.1) μg/dL, for LC/MS‐MS and ELISA method, respectively. CI, confidence interval; LC‐MS/MS, liquid chromatography‐tandem mass spectrometry.

One hundred and twenty older dogs met the inclusion criteria to establish the age‐specific RI. One remaining dog (15.5 years, 18.8 kg) was excluded because of renal azotemia (serum creatinine concentration 1.82 mg/dL [161 μmol/L]; USG 1.025). Fifty dogs included in the RI calculation had USG <1.030. Of these, 6 dogs had a MCS of 2/4; none was assigned a higher MCS. All of these 6 dogs had a serum creatinine concentration below the cutoff (median, 1.03; range, 0.67‐1.39 mg/dL). The dog with the highest serum creatinine concentration (1.39 mg/dL) had an ELISA SDMA of 14 μg/dL. This dog was monitored for 2 years in the longitudinal health study and despite never having well concentrated urine (USG >1.030), the dog did not develop renal azotemia and had stable mild muscle wasting. Age‐specific RI was 4.4 (90% CI, 3.7‐5.1) − 15.0 (90% CI, 14.3‐15.7) μg/dL and 6.4 (90% CI, 5.7‐7.2) − 17.4 (90% CI, 16.7‐18.1) μg/dL for LC/MS‐MS and ELISA method, respectively (Figure 6A,B).

## 4 DISCUSSION

Our results show that: (1) The LC‐MS/MS method is valid to measure SDMA and ADMA; (2) A significant difference between ELISA and LC‐MS/MS is present for SDMA measurements; and (3) The obtained age‐specific RI for older dogs for SDMA has a substantially higher upper limit for both methods compared to the general laboratory RI.

The LC‐MS/MS method used in our study reliably and precisely measured SDMA and ADMA in dogs and is therefore a valid gold standard method with the advantage of being commercially available and offered at a similar cost compared to the ELISA used in our study in Belgium. In our population of aged dogs, significantly higher SDMA results were observed with ELISA compared to LC‐MS/MS, despite a same upper limit of the laboratory RI (14 μg/dL). Although the mean difference was only 2.2 μg/dL, in 20% of dogs (n = 24/121) the difference exceeded 3 μg/dL which is a reported maximum acceptable measurement variation for SDMA concentrations in cats.^12^ Also, in a subset of dogs (8%, n = 9/118), the SDMA concentration was above the upper limit of the RI (>14 μg/dL) for ELISA, but still within the RI for the LC‐MS/MS method. Hence, the difference led to a different clinical interpretation of renal function. Using 16 or 18 μg/dL as an upper limit, as has been suggested in previous studies,^8^, ^10^ or using the age‐specific upper RI limit defined in our study, the clinical discrepancy between the 2 methods was overcome. This finding is also in agreement with the recent update of the International Renal Interest Society (IRIS) guidelines for SDMA (based on IDEXX SDMA) in staging patients with kidney disease. Until recently, a SDMA concentration >14 μg/dL (combined with other renal variables) was considered chronic kidney disease IRIS stage 2, but this concentration was adjusted to SDMA ≥18 μg/dL.^10^, ^32^ Increasing the cutoff could decrease the perceived benefit of SDMA being able to recognize decreased GFR more quickly compared to serum creatinine concentration.

Poor agreement between LC‐MS/MS and ELISA has been described for both ADMA and SDMA in human medicine.^6^, ^33^, ^34^ Overestimation of SDMA and ADMA concentrations by ELISA has been shown in humans,^6^, ^33^, ^34^ and this discrepancy is attributed to matrix dependence of the ELISA assay.^7^ Moreover, it is known that ELISA cannot distinguish L‐arginine from SDMA or ADMA, being an unique feature of mass spectrometry.^7^ For differentiation between sample groups, the LC‐MS/MS method therefore is considered much more reliable in human medicine.^7^ Results of a comparative study between LC‐MS/MS and the high throughput immunoassay (IDEXX ELISA) in dogs were never published in a peer‐reviewed journal.^16^, ^35^, ^36^ The higher SDMA concentrations with the ELISA method emphasize the importance of developing method‐specific RIs, rather than extrapolating from previous RIs.^5^, ^11^, ^28^ Transference should be avoided if a systematic difference (bias) or differences in analytical quality exist.^28^

To assess the cause for the difference observed, the effect of interfering substances (ADMA concentration, hemolysis, lipemia) on the SDMA concentration was evaluated. The ADMA concentration had no significant effect on the obtained SDMA concentration, although a weak positive relationship was noted. This finding was previously reported for LC‐MS/MS, but no data for the ELISA method were available.^5^, ^15^ In the previous study, absence of interference of arginine, monomethylarginine and homocitrulline on LC‐MS/MS SDMA also was shown. These variables were not further analyzed in our study, but could be interesting to explore further, especially arginine.^7^ The difference in SDMA concentration between the 2 methods decreased with increasing hemolysis. For lipemia, however, no significance was reached which could be related to the limited numbers of moderate to severely lipemic samples (Figure 5), possibly as a consequence of the unfed status of the dogs. A previous study has shown the robustness of LC‐MS/MS for SDMA measurement at increasing levels of hemolysis or lipemia.^5^ In 3 samples, SDMA could not be assessed by ELISA because of interfering lipemia or hemolysis, indicating errors when analyzing such samples by ELISA. Our data must be interpreted carefully, because only a few dogs had score 3 or 4 for hemolysis or lipemia and because of the study design, as it was not an interference study. However, it suggests that the ELISA method might not correctly measure SDMA in hemolytic samples.

The effect of PECCT measurement on the difference in SDMA concentration also was assessed in a subpopulation of dogs. Only a limited number of PECCT results were available (n = 14). As expected in this population of dogs without renal azotemia, all GFR results were within previously defined weight‐based RIs, which prevented us from calculating correlations between GFR and SDMA. Only a weak, nonsignificant trend toward a larger difference in SDMA between the 2 methods was observed for dogs with a lower filtration capacity (PECCT value; Figure 3). Although the median time interval between sampling for SDMA and PECCT measurement was 0 (range, 0‐6) weeks, intraindividual variability for SDMA could affect this result. Intraindividual CV of 14% previously was described over a short time period (6 weeks) for SDMA and was later confirmed in a long‐term study.^14^, ^37^ These findings suggest that SDMA concentration remains relatively consistent over time in dogs.^14^ In 11 cases (11/14, 79%), PECCT measurement was performed at the same timepoint as sampling for SDMA, and only in 2 and 1 instance, after 4 and 6 weeks, respectively. Overall, intraindividual variability seems to have little impact on the observed trend.

Overall, no obvious cause was found to explain the difference in SDMA concentrations between the 2 methods. Based on the human medical literature, the most likely remaining explanations are presence of matrix interference in the ELISA method and the superior specificity of LC‐MS/MS.^7^

Determining an age‐specific RI for this population of older dogs resulted in a substantially higher upper end of the RI, especially for ELISA compared to laboratory RIs (Figure 6A,B). Several studies in humans have shown a correlation between age and SDMA, suggesting the use of age‐adjusted cutoff values.^38^, ^39^, ^40^ However, this finding may be linked to the decrease in GFR associated with aging in humans.^40^ In adult dogs however only a limited effect of aging on GFR has been shown.^41^ In our study, because GFR was estimated only in a minority of dogs, an early stage of kidney disease in some included dogs cannot be completely excluded. Performing PECCT measurement is cumbersome and time‐consuming, and not routinely performed in practice. This limitation was minimized by excluding all dogs with renal azotemia. Moreover, doing so is common practice in a clinical setting. Because serum creatinine concentration is muscle dependent, assessment of muscle mass is important when screening for renal azotemia, especially in older dogs. Based on MCS in our study, misinterpretation of serum creatinine concentration seems unlikely. Two dogs had serum creatinine concentrations exceeding the upper limit of the laboratory RI (serum creatinine concentrations 1.82 and 1.93 mg/dL in combination with USG ≥1.030) and were not excluded from RI calculation. Although prerenal azotemia cannot be entirely excluded in these dogs, they did not show signs of dehydration or hypovolemia. One of these dogs had a normal GFR measurement, but in the other dog GFR was not available. Potential influence of prerenal azotemia on the reported SDMA concentrations was therefore considered negligible. Six dogs included in the RI calculation were on intermittent drug treatment, which may not be ideal for RI calculation. However, when recalculating the RI without these 6 dogs, we obtained the same 95% lower and upper RI limits for both methods (data not shown). The observed upper limit for SDMA in our population of older dogs is higher than a previously suggested cutoff (16 μg/dL) for the ELISA method to diagnose decreasing renal function. However, that study evaluated an adult population of dogs with median age of 5 (range, 2.5‐8.7) years.^8^ This finding confirms the need to develop age‐specific RIs for other laboratory variables, because age‐related but clinically insignificant changes more than likely also occur in other variables.^21^, ^22^

Our study had some limitations. First, we could not compare our age‐specific RI with a RI determined at the same institution using young and middle‐aged dogs. Also, only a limited number of giant breed dogs were included. As a result, the study population may not be representative of the entire geriatric dog population. However, SDMA is considered not to be dependent on lean body mass or breed (with the exception of Greyhounds), and thus the effect on the obtained RI likely is minimal.^42^, ^43^ The suggested RI ideally should be validated in a larger population of dogs, but our study showed that it is crucial to use a RI that is appropriate for the population that being investigated.

Previous studies indicate high analytical variability for the ELISA method.^11^, ^12^, ^13^ This variability could have affected our results, because individual sample analysis was done for ELISA. However, intra‐assay variability for the primary laboratory in this study was 5.18%, based on 499 samples with a mean SDMA of 12 ± 1 μg/dL (data provided by IDEXX bioanalytics, Vet Med Labor GmbH, Kornwestheim, Germany), suggesting minimal interference. Furthermore, for practical reasons, batch analysis was done for the LC‐MS/MS but not for the ELISA method, which could have influenced our results. However, recent data indicate that the influence of differences in storage conditions (eg, cooled transport overnight vs frozen) and storage time on SDMA is minimal.^13^ In that study, stability of SDMA for 7 days at 4°C (cooled) and 24 months at −80°C (frozen) was confirmed, indicating that it is unlikely that differences in storage condition were responsible for the observed difference between the 2 methods in our study.

In conclusion, the commercially available LC‐MS/MS method described in our study is a valid technique for SDMA measurement in canine serum. In this population of older dogs, significantly higher SDMA concentrations were obtained using ELISA compared to LC‐MS/MS indicating the need for method‐specific or device‐specific RIs. Lastly, substantially higher upper limits of the RI were found for SDMA when determining a RI in older dogs without renal azotemia for both methods, compared to laboratory RI.

## CONFLICT OF INTEREST DECLARATION

Greet Junius, Evi Van den Steen and Lisbeth Patteet are professionally affiliated with the commercial laboratory (SONIC Healthcare Benelux) that validated and performed the LC‐MS/MS analysis. They were not involved in study design, results processing, and statistical analyses of the acquired data. No other authors declare a conflict of interest.

## OFF‐LABEL ANTIMICROBIAL DECLARATION

Authors declare no off‐label use of antimicrobials.

## INSTITUTIONAL ANIMAL CARE AND USE COMMITTEE (IACUC) OR OTHER APPROVAL DECLARATION

The owners signed an informed consent, and the study was approved by the local and national ethical committees (EC 2019/39; DWZ/EV/19/115/75).

## HUMAN ETHICS APPROVAL DECLARATION

Authors declare human ethics approval was not needed for this study.
